# Predictors of Weight Change: Findings From an Employee Wellness Program

**DOI:** 10.3389/fendo.2019.00077

**Published:** 2019-02-19

**Authors:** Sue S. Feldman, Randyl A. Cochran, Tapan Mehta

**Affiliations:** Department of Health Services Administration, School of Health Professions, University of Alabama at Birmingham, Birmingham, AL, United States

**Keywords:** obesity, employee wellness, weight loss, readiness for change, health behavior

## Abstract

**Introduction:** Employers are instituting employee wellness programs that include educational, lifestyle coaching, and weight and other condition management components to address obesity-related issues in the workplace. However, the findings of such wellness initiatives have been mixed. The purpose of this exploratory study is to determine whether the readiness for change measures are important predictors of weight loss in an employee wellness program.

**Methods:** Retrospective data analysis of an employee wellness program conducted in the United States was conducted using data collected between 2014 and 2015 for people with BMI ≥ 30. These participants were assigned to one of two subprograms: weight management or condition management. We assessed the weight change within each program. Further, the relationship between weight change and readiness for change variables for weight, diet, and physical activity were examined by applying multiple linear regression and logistic regression models. The multivariable model included subprogram; gender; age; systolic and diastolic blood pressure; risk factor count; readiness for change for weight, activity, and diet; and stress level as covariates.

**Results:** There were 209 participants in the weight management program and 243 participants in the condition management program who met the criteria for obesity, resulting in a final sample of 452 participants. On average, the weight change for these participants was −0.28 pounds (*SD* = 15.55) and there was no statistical difference between the weight change in the two programs. When compared to the reference group (maintenance), participants at the action stage of physical activity, on average, lost weight (b = −4.59, *p* = 0.02). Likewise, participants at the pre-contemplation stage of physical activity lost weight when compared to the maintenance group (b = −26.24, *p* = 0.000). Participants at the pre-contemplation stage of physical activity had higher odds of achieving at least 5% weight loss than participants at the maintenance stage (OR = 5.80, *p* = 0.053).

**Conclusion:** Readiness for change for activity may be a predictor of weight change, and may predict the likelihood of achieving clinically significant weight loss. These findings can assist in targeting subjects for participation in such programs. The findings regarding the relationship between readiness for change and weight loss are counterintuitive, and further research is warranted in this area.

## Introduction

Obesity (BMI ≥ 30 kg/m^2^) prevalence has increased globally within the last four decades (1) and the United States has one of the highest rates of obesity (2). Furthermore, the economic burden of obesity is “considerable and rising” (3). The global economic impact of obesity is an estimated $2 trillion (US dollars), or 2.8% of gross domestic product (GDP) (4). Indirect societal costs of obesity are a result of increased absence from work and reduced productivity (4–6), as well as workplace injuries and disability payments (3, 4).

Employee wellness programs have become a popular mechanism to address health behaviors and reduce chronic conditions, including obesity, that impact workplace performance, and healthcare costs, especially for self-insured organizations (7). The workplace provides an ideal setting for employee wellness programs because nearly 60% of American workers receive their health insurance through their employer (8) and approximately 50% of waking hours are spent at work (9). The workplace also provides the necessary communication channels and social support for these programs to develop (10). For workers with obesity, in particular, employers pay more due to expenses related to medical claims, disability, and absenteeism (9). In the United States, over 37% of employees are considered overweight (they have a BMI in the range of 25–29.9 kg/m^2^), and 29% are classified as obese (6, 11, 12). Taken together, these factors provide an impetus for employers to take the lead in implementing programs to address obesity.

There is a general consensus on the need to reduce obesity rates, but developing and implementing effective strategies and policies has proven to be a difficult task (1). A RAND Health Quarterly study (5) examined various aspects of employee wellness programs, including the prevalence of these programs, how they are designed, their impact on health outcomes, the role of incentives, and factors that facilitate these programs. Employee wellness programs are common: approximately 50% of U.S. companies offer employee wellness programs (5, 13). These programs vary in their complexity, but most incorporate wellness screenings and interventions to educate participants about making healthy lifestyle choices (5). Many employee wellness programs offer incentives in order to increase participation (7, 10). These incentives come in various forms, including cash payments and discounts (7, 13). In addition to enhancing program participation, incentives can promote desirable outcomes, such as healthy eating and physical activity (13–15).

There is much debate surrounding the effectiveness of employee wellness programs (16). However, research indicates that the transtheoretical model (TTM), or the stages of change model, can be a useful framework for wellness programs that involve behavioral change (17–19). The transtheoretical model for change suggests that, “health behavior change involves progress through six stages of change: precontemplation, contemplation, preparation, action, maintenance, and termination” (20). Further, a recent randomized controlled trial found that there was no weight change in a behavioral weight loss program coupled with motivational interviewing compared to only the behavioral weight loss program (21). The authors indicated the need to tailor motivational interviewing according to the participant's baseline motivation to observe improvement in weight loss (21). Hence, in this exploratory study we will assess the average weight change observed in an employee wellness program and whether the baseline measure of readiness for change is a predictor of weight loss. In addition, we will assess whether other demographic and clinical factors are associated with weight loss.

The stages of change model is used to “explain and predict how and when individuals change behaviors” (17): it gauges individuals' readiness for change. Readiness for change emerges from the stages of change model (22), which is commonly used in the health promotion literature. Originally, the model was applied to smoking cessation, but it has been used to facilitate change in other behaviors, including diet and physical activity (23). According to the model, individuals are typically at different stages with regard to adopting and adhering to health behaviors. The model outlines five stages: (1) precontemplation (not thinking seriously about modifying current behavior); (2) contemplation (thinking about modifying current behavior); (3) preparation (finding the determination to modify current behavior; (4) action (changing habits and/or their environment); and (5) maintenance (successfully maintaining new habits and behaviors) (22). Based on this model, it is expected that individuals at earlier stages (e.g., precontemplation or contemplation) will be less likely to adopt healthier default behaviors than those in later stages (e.g., preparation and action) (24).

Studies have shown that the stages of change model can predict behavior modification in smoking cessation programs and, “to a lesser degree,” weight loss and maintenance programs (25). However, the model has received criticism. Sutton (26) acknowledged that finding significant differences in reported outcomes based on the participants' stage of change would support the model, but this approach ignores the possibility that different factors could have a more substantial effect at different stages (26, 27). A review of the literature on this model revealed that higher levels of self-reported readiness did not predict better treatment adherence, nor did it predict greater weight loss (28). Although the findings have been mixed, there are studies that support the use of interventions based on the stages of change model. For example, wellness interventions that accounted for participants' stage of change at baseline have been associated with increased physical activity (29, 30).

Physical inactivity is common among Americans, but interventions based on the stages of change model have been found to enhance physical activity in adults (30–32). However, Prochaska and DiClemente (33) have suggested that the model is cyclical rather than linear. Because individuals often fail to “establish and maintain lifestyle changes” (34, 35), it is common for them to regress back to an earlier stage of change (34). Research suggests that interventions based on the stages of change model are effective in initiating change in physical activity (34, 36). However, the effectiveness of these programs with regard to maintaining an active lifestyle is less clear: self-reported measures that gauge physical activity generally focus on short-term maintenance, but they fail to account for physical activity over longer periods of time (36). Other studies indicate that interventions developed from the stages of change model affect behavioral change rather than maintenance (34, 37).

Diet change is key to improving various health outcomes. Cummins et al. (38) connect the increase in obesity prevalence in the United States to “changes in the food system” (283); they suggest that interventions designed to reduce caloric intake and improve the quality of food that is consumed should be part of a larger effort to reduce obesity prevalence. However, many individuals are resistant to adopting healthy eating habits (39). Other factors, including personal routines and social norms, can complicate diet change (40, 41). As such, simply increasing access to more nutritional food options fails to produce the desired results with regard to health outcomes (38).

The literature references several barriers to healthy eating, including limited access to resources; lack of nutrition literacy; and other factors such as price, taste, and tradition that are often attached to food (42). Among a sample of adults with type 2 diabetes, individuals who were actively improving their diet perceived fewer barriers to making these changes than people who were at the preparation stage; often, the latter group becomes discouraged and reverts back to poor eating habits (39). A recent study evaluated the effectiveness of an intervention on various outcomes, including readiness to change dietary habits. Participants in the intervention group showed statistically significant progress in readiness for change between baseline and follow-up. The results further indicated that the improvement in dietary habits was significantly different between the intervention and control groups (43).

Determining individuals' readiness to lose weight is necessary, and designing interventions that are tailored to participants' level of readiness could result in successful weight loss and maintenance (44). Readiness for change can vary from one individual to another, even if these individuals share similar risk profiles. Ghannadiasl et al. (44) found that obese women in Iran were at different stages of readiness for change with regard to weight loss. Alakaam et al. (45) examined the relationship between individuals' perception of their weight and stage of change for weight loss; the findings revealed that participants who were classified as overweight or obese and who perceived themselves as such were more likely to be at the action stage with regard to weight loss. Similarly, in the Ghannadiasl et al. (44) study, obese women at the precontemplation stage of weight loss had a lower waist-to-hip ratio than women at other stages of change (44). For this exploratory study, we sought to determine whether the readiness for change measures for weight, physical activity, and diet are important predictors of weight loss in an employee wellness program.

## Materials and Methods

The program was designed to guide, support, and educate individuals in making necessary lifestyle changes for better health and condition management. One of the primary goals was for the participants to lose excess weight, which is associated with comorbidities. This section describes the employee wellness program and then discusses how the program participants were recruited and assigned to each of the four subprograms. Coaching techniques are briefly described. Finally, methods of statistical analysis are detailed.

### Employee Wellness Program Description

The employee wellness program that was used in this study is comprised of four subprograms: (1) low-risk, (2) lifestyle behavior, (3) weight management, and (4) condition management. In this paper we analyze data from the weight management and condition management programs only because these programs shared comparable factors: both were aimed at employees with obesity, both had similar age and gender distribution, and both had similar cardiometabolic risk profiles.

The primary area of focus for the weight management program is maintaining a healthy weight, which addresses issues related to obesity risk, portion control, water intake, caloric intake, and choosing healthy snacks. Similarly, the program encourages healthy eating: it promotes the Mediterranean diet, encourages participants to read food labels and use food trackers, and, when necessary, to consult with a dietitian. Additionally, the weight management program aims to assist participants with increasing physical activity (e.g., by developing a fitness routine and using fitness trackers), developing healthy sleep patterns, stress management, and smoking cessation.

The condition management program shares many components with the weight management program: these components include maintaining a healthy weight, healthy eating, increased physical activity, improved sleep patterns, stress management, and smoking cessation. In addition to these components, the condition management program promotes knowledge of various chronic conditions (diabetes, hypertension, and heart disease) and self-management of these conditions through practices such as blood glucose and blood pressure monitoring, knowledge and awareness of symptoms associated with these conditions, and developing an action plan for treatment. The condition management program also addresses issues related to medication adherence and encourages the use of self-monitoring devices, such as glucometers and blood pressure cuffs.

### Study Participants

Program participation was open to employees and their spouses who were beneficiaries of the employer-sponsored health insurance plan. Participants were recruited into the program through a variety of mechanisms: these included, but were not limited to emails, huddles, and flyers. The recruitment period for the program opened on September 16, 2013, and closed on December 13, 2013. Employees who were interested in the program accessed a website to register for participation. Program participants were paired with a coach, and they received phone calls during the 6 month engagement period (January 2014 to July 2014). Baseline measures were collected between April 1, 2014, and August 31, 2014. Post-intervention measures were collected between April 1, 2015, and August 31, 2015. All baseline and post-implementation measures (blood pressure, height, weight, waist circumference, HbA1c, and LDL) were collected by a third party vendor that was sub-contracted to perform measurements during employee wellness screenings. Because measurements were conducted by a third party vendor, the authors do not have access to the actual instruments that were used; however the vendor assured us that the same instruments for measure were used at both times and that instruments are calibrated in accordance with industry guidelines. It should be noted that risk of measurement error may occur between measurements.

An initial call gauged each participant's readiness for change (RFC) and set SMART goals (e.g., decrease in weight and waist circumference). The RFC instrument was included in the initial health assessment as completed by the employee and was derived from transtheoretical model (22) and included five of the six stages: precontemplation, contemplation, preparation, action, and maintenance. SMART goals are desired in behavior change based programs because they are Specific, Measureable, Achievable, Relevant, and Time-limited. In addition, each subprogram provided relevant educational resources for participants (e.g., various print, web, and electronic tools).

Based on a proprietary algorithm that considered BMI, HbA1c, LDL, systolic and diastolic pressures, and the American Heart Association My Life Check score, participants were stratified into low-, moderate-, and high-risk groups. Participants who were stratified into the low-risk group were assigned to the lifestyle management program. Participants stratified into the moderate- and high-risk groups were assigned to either the weight management program or the condition management program. These programs differed in that the condition management program primarily focused on teaching participants to manage diabetes, hypertension, and heart disease. The coaches employed motivational interviewing techniques to guide participants to adopt healthier default behaviors. For the purposes of the statistical analyses, we only examined participants who were classified as obese at baseline. All of these participants were either assigned to the weight management or condition management group.

Program participants consented to program participation as part of the program onboarding process. This paper used deidentified secondary data and was approved by the Institutional Review Board of University of Alabama at Birmingham IRB # 170421002.

### Statistical Analyses

The study conducted exploratory statistical analyses that included descriptive analyses, two-group comparisons, and multivariable regression models. While the statistical significance was at 0.05, we report the exact *p*-values, thus allowing the readers to interpret the findings based on their choice of multiple testing.

Descriptive statistics were generated for the outcome variables of interest for the weight management and condition management subprograms. These variables include weight, BMI, A1C levels, systolic and diastolic blood pressure, and waist circumference. In addition, gender and age composition for each of the subprograms were computed. A paired *t*-test was conducted to compare average weight for participants at baseline and at follow-up. Subsequently, we conducted separate paired *t*-tests within the weight management and condition management subprograms.

Next, we generated a variable for weight change: the difference between final weight and baseline weight was calculated. Welch *t*-tests were conducted in order to determine whether there was a significant difference in average weight change within the weight management and condition management subprograms. Then, linear regression models were run to determine predictors of weight change.

Finally, a binary variable was created to indicate whether participants met the criteria of clinically significant weight loss (46). “Clinically significant weight loss” refers to a loss of five percent or more of body weight and is expected to improve a number of health outcomes in individuals with obesity (47). Participants were assigned a value of 1 if they lost at least 5% of their baseline body weight. Otherwise, they were assigned a value of 0. A series of logistic regression models were run in order to determine factors that increase the likelihood that participants will attain clinically significant weight loss. Odds ratios for predictors of clinically significant weight loss are reported and interpreted in the Results section.

## Results

**Figure 1:**
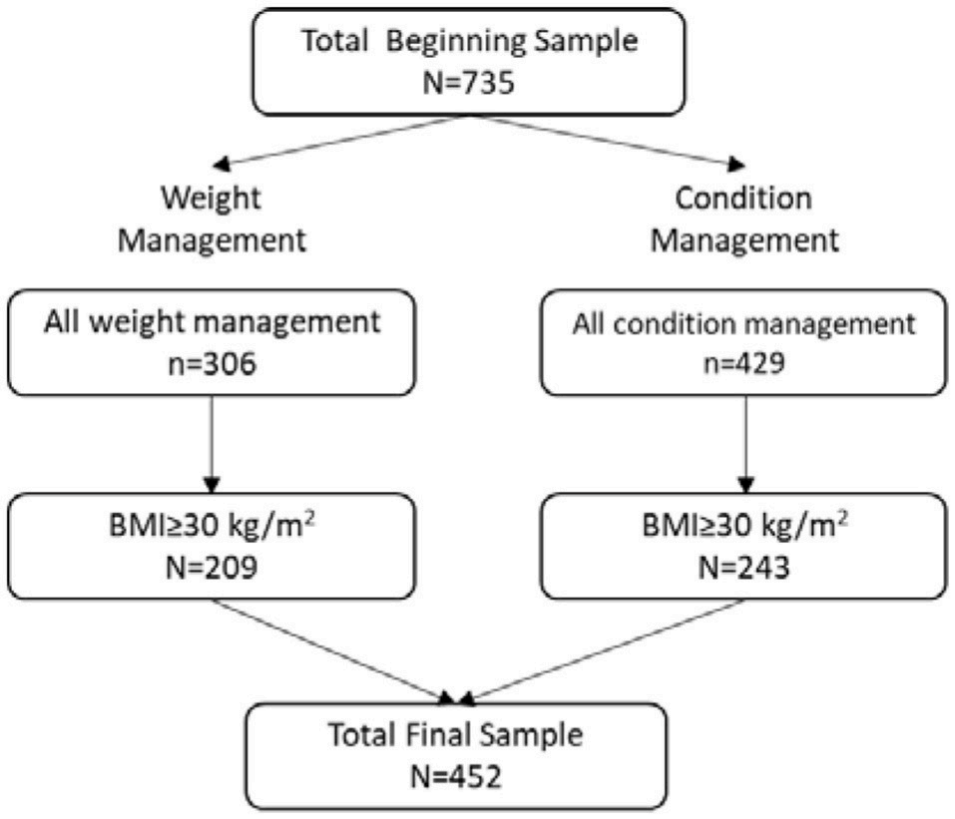
Study sample size flowchart.

A flowchart that depicts how the final sample size for this study was obtained is provided in Figure 1. Our total beginning sample size consisted of 735 participants across the weight management and condition management subprograms (Table 1), out of which 306 were enrolled at the start of the weight management program. Of those participants, 275 (89.87%) were female, and 31 (10.13%) were male. The average age of weight management program participants at baseline was 46.09 years (*SD* = 11.23); participant age ranged from 23 to 66 years. Analysis of baseline biometric data of all participants by program revealed no statistically significant difference in terms of BMI. However, there were significant differences in A1C, systolic, and diastolic blood pressure. Participants in the condition management group, on average, had higher A1C, systolic, and diastolic BP at baseline than participants in the weight management group. This finding is consistent with the proprietary algorithm used for participant program selection, as A1C and systolic and diastolic blood pressure were determinants for condition management vs. weight management.

**Table 1:** Baseline characteristics of participants.

	**Weight Mgmt. (*n* = 306)**	**Cond. Mgmt. (*n* = 429)**	**Total (*n*= 735)**
Age (years)	46.1 (11.2)	52.5 (9.9)	49.9 (10.9)
Gender	Male: 31 (10.13%)	Male: 63 (14.69%)	Male: 94 (12.79%)
	Female: 275 (89.87%)	Female: 366 (85.31%)	Female: 641 (87.21%)
A1C	5.5 (0.4)	5.9 (1.0)	5.7 (0.9)
Systolic BP	119.5 (12.8)	125.5 (15.8)	123.1 (15.0)
Diastolic BP	79.9 (9.4)	80.7 (9.9)	80.4 (9.7)
Weight (lbs.)	206.2 (39.7)	193.6 (44.8)	198.9 (43.2)
Waist circum. (in.)	40.0 (5.4)	38.8 (6.2)	39.3 (5.9)
BMI	34.2 (6.5)	32.3 (7.2)	33.1 (6.9)
BMI ≥ 30 kg/m^2^	*n* = 209	*n* = 243	*n* = 452

Next, the condition management program composition is examined. Four hundred twenty-nine participants were enrolled at the start of the condition management program. Three hundred sixty-six (85.31%) of the participants were female, and 63 (14.69%) were male. The condition management cohort was older than the weight management cohort: average age at the start of the program was 52.53 years (*SD* = 9.88). Participant age in the condition management group ranged from 23 to 76 years.

In terms of biometric measures, baseline measures for A1C, systolic and diastolic blood pressure, weight, waist circumference, and BMI were obtained at the initial screening. The means and standard deviations for these biometric measures (A1C, systolic and diastolic blood pressure, weight, waist circumference, and BMI), as well as age and gender composition, are reported below (see Table 1). These measures are reported for the weight management and condition management subprograms independently and in total.

For the purposes of the statistical analyses, we only examined participants who were classified as obese at baseline (BMI ≥ 30 kg/m^2^). This resulted in 209 participants in the weight management group and 243 participants in the condition management group. The total final sample was 452 participants. The RFC Diet level 4 had only one participant and therefore was excluded from the regression analyses.

### Weight Change Across Subprograms

The initial paired *t*-test compared baseline and final weight for participants in both subprograms. There were 451 paired observations. The average baseline weight was 219.65 pounds (*SD* = 37.14). Average weight after the intervention was 219.37 pounds (*SD* = 39.76). On average, participants who were classified as obese lost 0.28 pounds (*SD* = 15.55) over the course of the intervention. The weight loss was not statistically significant.

### Weight Management Program Outcomes

There were 209 paired observations in the weight management subprogram. The average baseline weight in this cohort was 219.02 pounds (*SD* = 38.75). Average weight after the intervention was 219.70 pounds (*SD* = 41.47). On average, participants in the weight management subprogram who were classified as obese gained 0.69 pounds over the course of the intervention; the weight gain was not statistically significant.

### Condition Management Program Outcomes

There were 242 paired observations in the condition management subprogram. The average baseline weight in this cohort was 220.19 pounds (*SD* = 35.76). The average final weight was 219.08 pounds (*SD* = 38.30). On average, participants in the condition management subprogram who were classified as obese lost 1.11 pounds over the course of the intervention; the weight loss was not statistically significant.

### Weight Change by Subprogram: Welch *t*-Test Results

The average weight change in the weight management subprogram was +0.69 pounds (*SD* = 15.66). The average weight change in the condition management subprogram was −1.11 pounds (*SD* = 15.44). We also compared whether the changes in weight differed across the two subprograms. The results of the Welch *t*-test, as well as the regression model (after adjusting for risk factors), indicate that there is no significant difference in weight change between the two subprograms (see Table 2 for the regression model).

**Table 2:** Predictors of weight change.

**Predictor**	**Coefficient (95% confidence interval)**	**t-stat**	***P*****-value**
Subprogram	−0.134 (−3.414, 3.145)	−0.08	0.936
Gender	−0.636 (−5.654, 4.382)	−0.25	0.803
Age	−0.088 (−0.237, 0.061)	−1.16	0.246
Systolic BP	−0.071 (−0.197, 0.054)	−1.12	0.262
Diastolic BP	−0.112 (−0.291, 0.068)	−1.22	0.223
Risk factor count	0.473 (−0.493, 1.438)	0.96	0.337
Number of observations = 417			

*A linear regression model was run to determine if any descriptive measures (gender and age), subprogram participation, and/or cardiometabolic factors are significant predictors of weight change. BP, blood pressure*.

### Predictors of Weight Change: Linear Regressions

Next, a series of linear regressions were conducted, in which weight change was the outcome (dependent variable). The initial model included subprogram (weight management or condition management); gender; age; various clinical measures; and readiness for change (RFC) variables for weight, physical activity, and diet as covariates. For our study, we considered five stages of change: maintenance (0); action (1); preparation (2); contemplation (3); and precontemplation (4). The reference category for the RFC variables was maintenance.

The results of the regression model are provided in Table 3. RFC for physical activity was a significant predictor of weight change. Participants at the action stage of physical activity lost weight when compared to the reference group (b = −4.59, *p* = 0.02), as did participants at the precontemplation stage of physical activity (b = −26.24, *p* = 0.000). Similar patterns emerged when we accounted for stress level in the regression model. Participants at both the action stage (b = −4.37, *p* = 0.029) and the precontemplation stage (b = −25.89, *p* = 0.000) of physical activity lost weight over the course of the intervention.

**Table 3:** Weight change predictors (regression model results).

**Predictor**	**Coefficients (95% confidence interval)**	**t-stat**	***P*****-value**
Subprogram	−0.379 (−3.669, 2.911)	−0.23	0.821
Gender	−1.855 (−6.951, 3.240)	−0.72	0.475
Age	−0.120 (−0.270, 0.029)	−1.58	0.114
Systolic BP	−0.076 (−0.202, 0.050)	−1.19	0.236
Diastolic BP	−0.099 (−0.276, 0.078)	−1.10	0.272
Risk factor count	0.711 (−0.326, 1.748)	1.35	0.178
RFC Weight (1: action stage)	0.517 (−7.970, 9.005)	0.12	0.905
RFC Weight (2: preparation stage)	−5.309 (−15.006, 4.387)	−1.08	0.282
RFC Weight (3: contemplation stage)	−3.629 (−17.902, 10.644)	−0.50	0.617
RFC Weight (4: precontemplation stage)	4.552 (−12.441, 21.546)	0.53	0.599
RFC Activity (1: action stage)	−4.592 (−8.453, −0.731)	−2.34	0.020
RFC Activity (2: preparation stage)	−2.856 (−7.832, 2.120)	−1.13	0.260
RFC Activity (3: contemplation stage)	−5.657 (−14.392, 3.078)	−1.27	0.204
RFC Activity (4: precontemplation stage)	−26.242 (−37.925, −14.561)	−4.42	0.000
RFC Diet (1: action stage)	3.578 (−1.323, 8.479)	1.44	0.152
RFC Diet (2: preparation stage)	3.884 (−2.709, 10.475)	1.16	0.247
RFC Diet (3: contemplation stage)	8.659 (−2.006, 19.325)	1.60	0.111
Number of observations = 403			

*This linear regression model is an extension of the analysis conducted in Table 2. In addition, we examined the RFC measures for weight, physical activity, and diet as predictors of weight change. BP, blood pressure; RFC, readiness for change. For this model, we had to exclude the RFC Diet (level 4, which is the precontemplation stage because of very small sample size and to allow the models to converge)*.

It is possible that baseline weight could be associated with subsequent weight loss. Taking this point into consideration, an additional linear regression model was run that included baseline weight as a predictor of weight change. Counter to intuition, baseline weight did not emerge as a significant predictor of weight change (b = −0.001, *p* = 0.951). To further compare the linear regression models with and without baseline weight included as a covariate, we generated the Akaike information criterion (AIC) to examine model fit. The AIC for the model without baseline weight was 3337.6; the AIC for the model with baseline weight included was 3339.6. The results of the sensitivity analysis indicate that including baseline weight does not enhance the model substantially and that our original findings are robust.

### Logistic Regression

Finally, a logistic regression model was run where the dependent variable was a binary variable that measured whether participants achieved clinically significant weight loss or not. Of the participants who were classified as obese, only 73 (16.19%) achieved clinically significant weight loss. The logistic regression model included gender, age, subprogram, clinical measures, and the readiness for change variables for weight, physical activity, and diet as covariates. Participants at the precontemplation stage of weight change (four observations) and diet change (one observation) were excluded from the analysis due to small sample size. The odds of clinically significant weight loss in participants at the precontemplation stage of physical activity were nearly six times higher than the participants at the maintenance stage; this finding approaches statistical significance (*OR* = 5.80, *p* = 0.053). These results are presented in Table 4.

**Table 4:** Predictors of clinically significant weight loss.

**Predictor**	**Odds ratio (95% confidence interval)**	**Z-statistic**	***P*****-value**
Subprogram	0.904 (0.489, 1.672)	−0.32	0.748
Gender	0.877 (0.328, 2.350)	−0.26	0.795
Age	0.993 (0.965, 1.021)	−0.49	0.628
Systolic BP	1.017 (0.994, 1.042)	1.43	0.153
Diastolic BP	1.010 (0.976, 1.044)	0.56	0.576
Risk factor count	0.968 (0.798, 1.175)	−0.33	0.745
RFC Weight (1: action stage)	0.854 (0.161, 4.545)	−0.18	0.854
RFC Weight (2: preparation stage)	2.378 (0.378, 14.960)	0.92	0.356
RFC Weight (3: contemplation stage)	2.152 (0.165, 27.989)	0.59	0.558
RFC Activity (1: action stage)	2.080 (0.924, 4.681)	1.77	0.077
RFC Activity (2: preparation stage)	1.181 (0.421, 3.309)	0.32	0.752
RFC Activity (3: contemplation stage)	2.631 (0.567, 12.201)	1.24	0.216
RFC Activity (4: precontemplation stage)	5.802 (0.976, 34.487)	1.93	0.053
RFC Diet (1: action stage)	0.511 (0.210, 1.248)	−1.47	0.141
RFC Diet (2: preparation stage)	0.452 (0.138, 1.476)	−1.32	0.188
RFC Diet (3: contemplation stage)	0.328 (0.045, 2.419)	−1.09	0.274
Number of observations = 399			

*The logistic regression model was run to determine whether there are any significant predictors of clinically significant weight loss (5% or more of original weight). BP, blood pressure; RFC, readiness for change*.

Similar to the linear regression models, we ran an additional logistic regression model to test the idea that baseline weight could have an association with the likelihood that an individual would achieve clinically significant weight loss. Again, baseline weight did not emerge as a significant predictor of clinically significant weight loss (OR = 0.9997, z = −0.07, *p* = 0.947). Again, we generated AIC values for the logistic regression models with and without baseline weight included as a covariate to examine model fit. The AIC for the logistic regression model without baseline weight was 371.2; the AIC for the model with baseline weight included was 373.2. Again, the results of the sensitivity analysis indicate that the model was not enhanced by the inclusion of baseline weight as a predictor, and our original findings are robust.

## Discussion

This study estimated the average weight changes in an employee wellness intervention who were classified as obese, and compared these weight changes between two programs: weight management and condition management. The average weight loss for participants in both the weight management and condition management subprograms was 0.28 pounds. With regard to weight loss, the results of this employee wellness program are consistent with other published findings (21, 43). Specifically, Mache et al. (43) found that during a 12 month workplace intervention, the average weight loss was 0.5 kilograms, or approximately 1.10 pounds. Additionally, only 7% of the intervention group achieved clinically significant weight loss; an additional 3% lost at least 10% of their original body weight (43).

Further, the study explored whether RFC measures were predictors of weight change. The RFC measures for physical activity may help to predict weight change. The findings related to the physical activity of readiness for change will be discussed in the following paragraphs.

Participants at the action and precontemplation stages of change lost weight when compared to those at the maintenance stage. Based on the literature, the former is to be expected. Macchi et al. (48) propose the following: “Applied to weight management, weight loss occurs during the action stage and weight-loss maintenance occurs during the maintenance stage” (48).

With regard to participants at the precontemplation stage, the findings are counterintuitive. Individuals at more advanced stages of change are more likely to enroll in and complete workplace physical activity challenges (49, 50). Programs that do not increase physical activity gradually could deter participation from individuals at lower stages of change (e.g., precontemplation and contemplation). Tsai et al. (28) offer a possible explanation for this finding: “Individuals may overestimate their readiness because they do not clearly understand what behaviors are needed to make them successful, or because they greatly desire the outcome of weight loss” (28). From this perspective, it is plausible that participants who report higher levels of readiness for change might fail to achieve the desired results, and those at earlier stages might actually report better outcomes at the end of the intervention. However, additional research is needed to understand the weight change within the precontemplation group.

Every attempt was made to address or mitigate limitations. However, this study is limited in terms of participant recruitment, as the sample was heavily skewed toward females and did not include a control group. Because the study utilizes observational data, it is an exploratory study; we are unable to make any generalizations about causal relationships between participants' stage of change and weight loss. Moreover, testing of multiple hypotheses as part of an exploratory study can lend to false discoveries and it may be that RFC on physical activity may be a false positive. We provide exact *p*-values so that the readers can use their preferred approach and interpretation with respect to multiple testing. Another limitation of the study is that it only accounts for baseline and final (post-intervention) measures. A more useful approach would be to collect data (e.g., weight, blood pressure, waist circumference) from study participants at multiple time points, similar to the Moss et al. (21) study. For this study, we collected self-reported data for the RFC measures. The use of self-reported data introduces the possibility of various biases, the most likely of which is social desirability bias. When social desirability bias is present, participants report inaccurately in an effort to present themselves in a positive light. The presence of bias limits the validity of our results and could partially explain the counterintuitive findings of the study.

In addition to potential validity issues, the exploratory nature of the study and the use of self-reported data could restrict the generalizability of our findings (51). Even with the limitations, this study provides valuable information for group wellness programs as employers begin to think about offering wellness programs to employees.

It is worth noting that this study did not compare the costs of the program to savings generated from reduced absenteeism.

Future research would benefit from following participants over a longer period of time, including a more rigorous design that includes a control group, ideally randomized controlled studies, and more objective and measured measures to address the issues of bias, measurement error, and generalizability. Pragmatic designs, including Sequential Multiple Assignment Randomized Trial (SMART) designs that can be used to develop adaptive interventions, need to be leveraged in developing effective wellness programs (52, 53). Finally, analyses such as cost-effectiveness, cost-benefit, and miscrosimulations can be used to evaluate the short-term and long-term economic impact of wellness programs.

## Conclusion

This study highlights the importance of considering readiness for change within physical activity as a potential predictor of weight change. There is at least a moderate level of evidence that suggests use of the stages of change model as a framework for employee wellness interventions and more research in this area is warranted to test this hypothesis using a more rigorous design.

## Author Contributions

SF conceived the design of the study, conducted data collection, contributed to data interpretation, and provided overall direction and planning. RC contributed to the data analysis. TM provided oversight for data analysis and interpretation activities. SF, RC, and TM contributed to the manuscript writing, editing, and revising.

### Conflict of Interest Statement

The authors declare that the research was conducted in the absence of any commercial or financial relationships that could be construed as a potential conflict of interest.
